# Capturing daily well-being: psychometric evaluation of the MHC-SF in a daily diary design

**DOI:** 10.3389/fpsyg.2026.1887563

**Published:** 2026-07-28

**Authors:** Veronika Ploke, Bernad Batinic, Stefan Stieger

**Affiliations:** 1 Faculty of Psychology, Karl Landsteiner University, Krems an der Donau, Austria; 2 Department of Work, Organizational and Media Psychology, Johannes Kepler University Linz, Linz, Austria

**Keywords:** daily well-being, experience sampling method, generalizability theory, MHC-SF, psychometrics

## Abstract

Research on well-being has predominantly relied on cross-sectional retrospective person-level assessments, which may overlook short-term fluctuations across contexts and time. The present study evaluated a daily diary adaptation of the Mental Health Continuum-Short Form (MHC-SF) in a 14-day experience sampling design comprising 13 end-of-day assessments followed by a final questionnaire on day 14 in a German-speaking sample (*N* = 262). The retrospective two-week MHC-SF and the daily adaptation of the MHC-SF were examined using traditional psychometric approaches (internal consistency, confirmatory factor analysis) and advanced methods (generalizability theory, multilevel reliability, multilevel confirmatory factor analysis, and exploratory structural equation modelling). Results provided preliminary and qualified support for the daily diary adaptation of the MHC-SF. Evidence was strongest for the total MHC score and emotional well-being, which showed comparatively greater within-person variability and acceptable within-person reliability. In contrast, psychological and especially social well-being showed higher between-person stability and weaker evidence for capturing short-term daily fluctuations. The retrospective two-week MHC-SF showed excellent internal consistency, but its factor structure was not well captured by restrictive CFA models. ESEM provided better global fit, although the loading pattern suggested a dominant general well-being factor and limited empirical separation of the three domains, particularly for social well-being items. Subsequent lagged analyses indicated moderate day-to-day stability of well-being. Overall, these results support the utility of the MHC-SF for assessing overall and emotional aspects of daily well-being in ecologically valid settings, whereas psychological and particularly social well-being should be interpreted more cautiously and may require further adaptation for intensive longitudinal designs.

## 1 Introduction

It is a fact widely acknowledged in well-being research that a multitude of questionnaires has been developed to capture this complex construct (Lindert et al., 2015; Linton et al., 2016). Despite differences in approaches and operationalizations, most well-being instruments are rooted in two dominant traditions: the hedonic perspective, which focuses on positive emotions and life satisfaction (Diener et al., 2009; McDowell, 2010), and the eudaimonic perspective, which emphasizes psychological functioning, meaning and personal growth (Ryff and Keyes, 1995). While some instruments capture a single aspect of well-being, either hedonic (e.g., The Satisfaction with Life Scale; SWLS; Diener et al., 1985) or eudaimonic (e.g., The Psychological Well-Being Scale; Ryff, 1989), others integrate both perspectives, such as the PERMA-Profiler (Butler and Kern, 2016) and the Mental Health Continuum-Short Form (MHC-SF; Keyes, 2002, 2005). Among these, the MHC-SF is widely used due to its brevity (14 items) and extensive empirical validation across diverse cultural contexts (e.g., a 38-nation study: Żemojtel-Piotrowska et al., 2018).

Conceptually, the MHC-SF is based on Keyes’s (2002) two-continua model, which considers mental health and mental illness to be related yet distinct concepts. A notable strength of the MHC-SF is that it explicitly incorporates social well-being as a separate dimension, alongside emotional and psychological well-being. This allows for a more comprehensive evaluation of mental health than approaches focusing primarily on hedonic or individual psychological aspects of well-being (Lamers et al., 2011). Specifically, it captures three dimensions: emotional well-being (EWB; happiness, interest in life and life satisfaction), social well-being (SWB; social contribution, integration, actualization, acceptance and coherence), and psychological well-being (PWB; self-acceptance, environmental mastery, positive relations with others, personal growth, autonomy and purpose in life).

A substantial body of research has demonstrated the MHC-SF’s good psychometric properties (e.g., Iasiello et al., 2022; Lamers et al., 2011) and it has since been widely used in international research (e.g., Chinese: Guo et al., 2015; French: Doré et al., 2017; German: Żemojtel-Piotrowska et al., 2018; Stieger et al., 2023b, 2024). However, its factor structure has shown inconsistent replication across samples and contexts. For example, a 38-nation study (Żemojtel-Piotrowska et al., 2018) supported the three-factor structure over a one-factor structure solution in most cases but also highlighted variability in model fit across contexts.

Although the MHC-SF has been used in many studies to date, it has predominantly been applied in cross-sectional or long-term longitudinal studies with few measurement occasions (e.g., Lamers et al., 2011). Such designs provide static snapshots and may therefore not adequately capture the dynamic nature of well-being, which fluctuates within individuals over time (Curran and Bauer, 2011; Sonnentag, 2015; Stieger and Reips, 2019). Intensive longitudinal methods such as the Experience Sampling Method (ESM; Bolger et al., 2003; Csikszentmihalyi and Larson, 1987) are particularly valuable for capturing these temporal dynamics (Wichers et al., 2015). By measuring directly within individuals’ daily lives, these approaches reduce recall bias and allow for the differentiation between stable between-person differences and within-person variability (Eisele et al., 2021; Schimmack et al., 2010).

To date, only a limited number of studies have adapted the MHC-SF for daily assessment. The original retrospective version uses a six-point Likert scale (0 = *never* to 5 = *every day*) with a one-month reference period. In contrast, daily adaptations focus on shorter time frames. For example, a binary response format (0 = *never*; 1 = *at least once*) was found to have good within-person reliability (*R_c_* = 0.67–0.76; using the approach of Shrout and Lane, 2012) and excellent between-person reliability (*R_kR_* = 0.98–0.99) in a four-week ESM study (Stieger et al., 2023a). A subsequent study (Stieger et al., 2023b) using a four-point scale (from 0 = *never* to 3 = *three or more times*), reported comparable results (*R_kR_* = 0.94–0.98; *R_c_* = 0.60–0.87), along with expected associations with trait mental health and somatic symptom burden.

While these findings are promising, systematic psychometric evaluation of the MHC-SF across both daily and retrospective person-level assessments in a longitudinal context remains limited. The aim of this study is to investigate whether an internationally recognised trait instrument can be adapted for use in a daily diary format, and to extend a well-established, theory-driven measure to intensive longitudinal research.

### 1.1 Research questions

The following research questions were addressed to evaluate the psychometric performance of the MHC-SF as a retrospective person-level and daily measure within a 14-day interval-contingent ESM design:

1. Does the MHC-SF demonstrate adequate reliability and factorial structure when used as both a retrospective person-level measure and as a daily measure?
2. How are daily MHC-SF scores associated with retrospective person-level MHC-SF scores and selected exploratory indicators?
3. To what extent do emotional, social, and psychological well-being exhibit within-person variability and temporal stability over the daily assessment phase?

## 2 Methods

This study used a 14-day interval-contingent ESM design (Reis and Gable, 2000), with assessments scheduled once per day at predefined end-of-day intervals. This design comprised 13 end-of-day assessments and a final questionnaire on day 14 to evaluate daily and retrospective person-level well-being.

### 2.1 Participants and data cleaning

To reach a broad sample of adults, participants were recruited between April and August 2022 via various informal and community-based channels. These strategies included social media posts on platforms such as Instagram and Facebook, as well as outreach through personal networks and word of mouth. In total, 376 individuals completed the baseline demographic questionnaire. Of these, 271 also completed the final questionnaire at the end of the study, which was required for inclusion, as all retrospective person-level measures (MHC-Trait, SSS-8-Trait, VAS-Trait) were administered exclusively at this time.

Data quality was analysed by comparing demographic variables collected at baseline and at the end of the study. Implausible entries (e.g., 221 years or ‘1971’ instead of the correct age) were corrected if unambiguous and otherwise removed. Two participants under the age of 18 were excluded, and duplicated user entries were resolved by retaining the first entry. In line with previous ESM research (Eisele et al., 2021; van Berkel et al., 2019), responses submitted after midnight were excluded as the reference day was ambiguous.

Following data cleaning, the final sample comprised 262 participants with a mean age of 34.95 years (*SD* = 14.93, range = 18 to 78; 3% missing). Most participants identified as women (67%; 31% men; 1% non-binary; 1% missing). Regarding current relationship status, most participants reported being in a relationship (33%; 32% married/civil union; 26% single; 3% divorced; 1% widowed; 5% missing). Most participants were from Austria (79%; 13% Germany; 7% other countries; 1% missing).

### 2.2 Measures

#### 2.2.1 Mental health continuum-short form

The Mental Health Continuum-Short Form (MHC-SF; Keyes, 2002, 2005, 2018; German version: Żemojtel-Piotrowska et al., 2018) is a 14-item questionnaire that assesses well-being across three dimensions: emotional well-being (EWB), psychological well-being (PWB), and social well-being (SWB). In the present study, two versions were used. First, the retrospective two-week version of the MHC-SF, hereafter referred to as MHC-Trait was administered at the end of the study. To align with the study duration, the original four-week timeframe was adapted to the same period covered by the daily assessments (“*When thinking back over the past two weeks, how often did you feel…*”). Items were rated on the original six-point Likert-type scale ranging from 0 (*never*) to 5 (*every day*). The term MHC-Trait is therefore used to distinguish this retrospective person-level assessment from the daily ratings, rather than to imply a pure baseline dispositional trait measure. The daily version (hereafter MHC-State) was adapted for end-of-day assessment (see also Stieger et al., 2023b). Items referred to well-being during the current day (“*During today, how often did you feel…?*”) and were rated on a four-point Likert-type scale ranging from 0 (*never*) to 3 (*three or more times*).

#### 2.2.2 Somatic Symptom Scale-8

The Somatic Symptom Scale-8 (SSS-8; German version: Gierk et al., 2014) is a brief version of the Patient Health Questionnaire-15 (PHQ-15; Kroenke et al., 2002). It assesses somatic symptom burden across eight common physical complaints, including pain, cardiopulmonary symptoms, and fatigue. The SSS-8 has demonstrated good reliability and validity in both clinical and general populations (Gierk et al., 2014; Kliem et al., 2021). The trait version (hereafter SSS-8-Trait) was administered once at the end of the study using a two-week reference period (“*How strongly did you feel affected by the following complaints during the past 14 days?*”). Items were rated on a five-point scale ranging from 0 (*not at all*) to 4 (*very much*). The daily version (hereafter SSS-8-State) used the same items and response format as the retrospective version but referred to symptoms experienced during the previous night and the current day (“*How strongly did you feel affected by the following complaints last night and today?*”).

#### 2.2.3 Visual Analogue Scale (VAS) of well-being

Following previous ESM research (Stieger et al., 2021), a one-item Visual Analogue Scale of well-being (VAS; Griffiths and Stefanovski, 2019) was incorporated to provide participants with personalized graphical feedback in the ESMira app. In the present analyses, the VAS was used as an exploratory single-item indicator of subjective well-being. The trait version (hereafter VAS-Trait) was administered at the end of the study (“*How would you rate your average well-being during the past two weeks?*”), with responses ranging from 1 (*very poor*) to 10 (*very good*). The daily version (hereafter VAS-State) was administered at the end of each day (“*How would you rate your average well-being today?*”), with responses ranging from 1 (*very poor*) to 100 (*very good*).

### 2.3 Procedure

Data were collected via the ESMira smartphone app (Lewetz and Stieger, 2024), which enables assessments in participants’ natural environments. After providing informed consent, participants completed a baseline questionnaire assessing demographic variables (age, gender, current relationship status, and nationality). During the daily assessment phase, participants received 13 end-of-day notifications at their chosen time (default: 8 p.m.). If no response was recorded, a reminder message was sent after 15 min. At the end of the study, participants completed the demographic questionnaire again to assess consistency, along with the retrospective person-level versions of all measures. Participants were also invited to provide optional feedback about the study. To enhance engagement and compliance, the ESMira app provided real-time graphical feedback on participants’ individual well-being data (see Supplementary Figure S1) and offered an anonymous chat function to contact the study team. To minimise participant burden (Eisele et al., 2020, 2021), the daily questionnaire included brief measures of well-being (MHC-State), somatic burden (SSS-8-State), and a visual analogue scale of well-being (VAS-State).

### 2.4 Statistical analyses

#### 2.4.1 Cross-sectional analyses of the retrospective two-week MHC-SF

For the retrospective MHC-SF, reliability was assessed using Cronbach’s α. The factor structure was examined using confirmatory factor analyses (CFAs) with robust maximum likelihood estimation (MLR), implemented in the *lavaan* package in *R* (Rosseel, 2012). Model fit was evaluated using commonly accepted thresholds (χ^2^/*df* < 3.0, RMSEA < 0.06, SRMR < 0.09, CFI ≥ 0.95; Hu and Bentler, 1999; Satorra and Bentler, 2001; Steiger, 2007). To evaluate alternative representations of the scale structure, one-factor, two-factor, correlated three-factor, and second-order CFA models were estimated for MHC-Trait. As the MHC total score was used in subsequent analyses, a second-order CFA was additionally estimated, with emotional, social, and psychological well-being loading on a higher-order well-being factor.

In line with recent recommendations for multidimensional well-being measures (Joshanloo, 2022; Marsh et al., 2013, 2020; Morin et al., 2020; Żemojtel-Piotrowska et al., 2018), exploratory structural equation modelling (ESEM; Asparouhov and Muthén, 2009) with GeominT rotation was conducted. GeominT rotation was chosen because it approximates a simple loading structure while allowing small, non-zero cross-loadings and correlated factors, which is appropriate for multidimensional psychological constructs such as well-being (Asparouhov and Muthén, 2009; Marsh et al., 2013). ESEM was applied as a complementary approach to examine whether the poorer fit of the CFA model reflected the restrictive assumption of zero cross-loadings rather than a fundamental misspecification of the three-dimensional factor structure. Because the primary CFA and ESEM models were estimated using different estimation procedures, an additional model comparison was conducted in *lavaan* using MLR estimation for both models.

#### 2.4.2 Longitudinal analyses of MHC-state

To account for the nested data structure, in which daily observations were nested within individuals, multilevel confirmatory factor analysis (MCFA; Geldhof et al., 2014) was performed using the lavaan package (Rosseel, 2012). Since conventional fit indices such as the RMSEA and CFI were developed for single-level models and may be less sensitive to misspecifications at the between level (Hsu et al., 2015; Huang, 2017), within-level SRMR-W and between-level SRMR-B were reported alongside the conventional fit indices.

Given the four-point ordinal response format of the MHC-State items, the model was additionally estimated as a two-level MCFA with weighted least squares mean- and variance-adjusted estimation (WLSMV; Muthén et al., 1997), in line with methodological discussions on CFA with ordinal indicators (Brauer et al., 2023). This analysis was conducted in *Mplus* (Muthén and Muthén, 1998–2017), with all 14 MHC-State items specified as ordered categorical indicators. Emotional, social, and psychological well-being were specified as correlated latent factors at both the within- and between-person levels.

Generalizability Theory coefficients (G-theory; Cronbach et al., 1963) were computed using the *gtheory* package in *R* (Moore, 2016) to assess the consistency of MHC-State scores across repeated measurements. Unlike classical test theory, which typically summarises measurement error as a single residual component, G-theory allows multiple sources of measurement error to be estimated simultaneously across repeated measurements (Brennan, 2011; Geldhof et al., 2014). Additionally, multilevel reliability estimates were computed using the *psych* package (Revelle, 2026) to distinguish reliability at the between-person and within-person levels. *R*_kR_ was used to estimate the reliability of between-person differences, whereas *R*_c_ was used to estimate the reliability of within-person fluctuations across days.

Scale sum scores were used for state-level descriptive statistics and mixed-effects models involving MHC-State and SSS-8-State, whereas retrospective person-level descriptives were reported as scale mean scores to retain the original response metric. Two-level regression models were estimated using the *lme4* package (Bates et al., 2015), *lmerTest* (Kuznetsova et al., 2017), and *sjstats* package (Lüdecke, 2018), with daily measures at Level 1 nested within individuals at Level 2. Null models were computed to partition variance into within- and between-person components (ICC). Item-level ICCs were estimated to determine between- and within-person variance components for each MHC-State item. Linear mixed models with day as a predictor were computed to examine changes in well-being over the assessment period. Additional models evaluated whether retrospective MHC-Trait moderated changes in MHC-State over time by including the Day × MHC-Trait interaction. To support the time-trend analyses, lagged multilevel models were estimated in which MHC-State on a given day was predicted by MHC-State on the previous day.

## 3 Results

### 3.1 Dropout analysis and data quality

Of the participants who completed the baseline demographic questionnaire, 33% did not complete the end-of-study questionnaire. Dropout analyses showed that participants who dropped out were significantly younger (*M* = 30.30, *SD* = 14.77) than completers (*M* = 34.88, *SD* = 14.89), *t* (368) = −2.74, *p* = 0.01, *d* = −0.31. No significant differences emerged for gender [χ^2^ (2) = 4.96, *p* = 0.08, *φ* = 0.12], relationship status [χ^2^(4) = 5.07, *p* = 0.28, φ = 0.12], or nationality [χ^2^(2) = 1.84, *p* = 0.40, φ = 0.07]. In the longitudinal analyses, early dropouts reported lower MHC-State values, but this difference was not significant [*b* = −1.13, 95% CI (−2.39, 0.10), *p* = 0.08]. The interaction Day × Dropout was also not significant [*b* = −0.02, 95% CI (−0.04, 0.00), *p* = 0.08].

During the daily assessment period, participants could submit a maximum of 3,406 end-of-day reports (13 prompts × 262 participants). Following data cleaning, 2,778 valid end-of-day reports were retained, corresponding to an overall compliance rate of 80%. On average, participants completed 10.39 out of 13 daily reports (*SD* = 2.64; range = 2–13). Demographic variables showed a high level of stability across the two measurement occasions. Age showed almost perfect stability (*r* = 0.99, *p* < 0.001). Gender (men, women, non-binary) also remained highly consistent (*κ* = 0.96, *p* < 0.001), with only three participants changing their reported category. Relationship status showed slightly more variability, with 12 participants reporting a change (e.g., from *single* to *in a relationship*), but still demonstrated strong consistency (contingency coefficient = 0.90, *p* < 0.001).

### 3.2 Results of the retrospective two-week MHC-SF (cross-sectional)

#### 3.2.1 Item-level descriptive statistics and reliability

Internal consistency was high across retrospective person-level measures, with Cronbach’s α ranging from 0.83 to 0.93 (see Table 1).

**Table 1:** Means, standard deviations, reliabilities, and intercorrelations of retrospective person-level variables.

Scale (# Items)	M (SD)	(1)	(2)	(3)	(4)	(5)	(6)
(1) MHC (14)	3.23 (1.02)	0.93					
(2) MHC EWB (3)	3.63 (1.05)	0.87***	0.88				
(3) MHC PWB (6)	3.53 (1.08)	0.94***	0.82***	0.88			
(4) MHC SWB (5)	2.62 (1.23)	0.89***	0.65***	0.70***	0.85		
(5) SSS-8 (8)	0.73 (0.95)	−0.35***	−0.42***	−0.32***	−0.27***	0.83	
(6) VAS (1)	7.26 (1.58)	0.60***	0.67***	0.55***	0.48***	−0.55***	-

*N* = 262. Means and standard deviations are reported as scale mean scores for the retrospective two-week measures. MHC, mental health continuum-SF, range 0–5; MHC EWB, emotional well-being subscale, range 0–5; MHC PWB, psychological well-being subscale, range 0–5; MHC SWB, social well-being subscale, range 0–5; SSS-8, Somatic Symptom Scale-8, range 0–4; VAS, Visual Analogue Scale of well-being, range 1–10. Diagonal entries represent Cronbach’s α. ***p < 0.001.

At the item level, retrospective MHC-SF items showed positive inter-item correlations (*r* = 0.28 to 0.89), supporting the internal consistency of the scale (α = 0.94; see Supplementary Table S1).

#### 3.2.2 Confirmatory factor analysis (CFA) and exploratory structural equation modelling (ESEM)

The results of the CFAs and ESEM models of the retrospective MHC-SF are summarised in Table 2.

**Table 2:** Results of confirmatory factor analyses and ESEM of the retrospective two-week MHC-SF.

Model	χ^2^(df)	χ^2^/df	CFI	RMSEA (90% CI)	SRMR
CFA one-factor model	530.42 (77)	6.89	0.76	0.15 (0.14–0.16)	0.09
CFA two-factor model	297.26 (76)	3.91	0.88	0.11 (0.10–0.12)	0.12
CFA three-factor model	270.54 (74)	3.66	0.90	0.10 (0.09–0.11)	0.12
ESEM two-factor model	135 (66)	2.05	0.99	0.06 (0.05–0.08)	0.04
ESEM three-factor model	81.86 (55)	1.49	0.99	0.04 (0.02–0.06)	0.03

CI, confidence interval; CFA, confirmatory factor analysis; ESEM, exploratory structural equation modelling. The three-factor models included EWB, PWB, and SWB as separate factors. The two-factor models combined EWB and PWB and retained SWB as a second factor. CFA models were estimated using robust maximum likelihood estimation; for these models, χ^2^ values refer to robust scaled test statistics. ESEM models were estimated using GeominT rotation.

Based on theoretical expectations (Keyes, 2002, 2005), the original three-factor CFA model comprising emotional, psychological, and social well-being was tested. The model showed suboptimal fit (χ^2^/df = 3.66; CFI = 0.90; RMSEA = 0.10; SRMR = 0.12). Neither the one-factor model nor the two-factor model combining EWB and PWB achieved acceptable model fit (CFIs < 0.90, RMSEA > 0.11). Thus, the CFA results did not provide sufficient support for a simple factor structure with cross-loadings constrained to zero. The second-order CFA showed the same global fit as the correlated three-factor CFA, χ^2^(74) = 270.54, CFI = 0.90, RMSEA = 0.10, SRMR = 0.12, but produced a negative residual variance for psychological well-being and was therefore interpreted with caution.

In a next step, ESEM models were estimated as a more flexible alternative to CFA. The three-factor ESEM model showed the best fit among the tested retrospective person-level models (χ^2^/df = 1.49; CFI = 0.99; RMSEA = 0.04; SRMR = 0.03). However, inspection of the loading pattern suggested that this improved fit did not reflect a clear three-domain structure. Instead, almost all items loaded substantially on the first factor, indicating a strong general well-being dimension (see Supplementary Table S3). The remaining factors primarily captured specific variance among selected social well-being items and were therefore interpreted cautiously rather than as distinct emotional, social, and psychological well-being dimensions.

### 3.3 Results of the MHC-state (longitudinal data)

#### 3.3.1 Descriptive analyses

Generalizability analyses (G-theory) indicated high reliability of MHC-State over time and across items (see Table 3).

**Table 3:** Means, standard deviations, reliabilities, and intercorrelations of state-level variables.

Scale (# Items)	M (SD)	(1)	(2)	(3)	(4)	(5)	(6)
(1) MHC (14)	22.87 (9.12)	0.93	0.89	0.96	0.91	−0.27	0.55
(2) MHC EWB (3)	5.94 (2.07)	0.76	0.87	0.85	0.71	−0.31	0.59
(3) MHC PWB (6)	10.64 (4.20)	0.87	0.51	0.90	0.79	−0.25	0.52
(4) MHC SWB (5)	6.30 (3.56)	0.82	0.46	0.54	0.88	−0.21	0.47
(5) SSS-8 (8)	4.45 (3.62)	−0.17	−0.16	−0.15	−0.11	0.22	−0.64
(6) VAS (1)	66.05 (16.22)	0.53	0.52	0.45	0.36	−0.44	-

*N* = 262, *k* = 2,778 available daily observations; pairwise sample sizes varied slightly due to missing values. Means and standard deviations for MHC-State and SSS-8-State are reported as scale sum scores. MHC, mental health continuum-SF (State; range 0–42); MHC EWB, emotional well-being subscale (State; range 0–9); MHC PWB, psychological well-being subscale (State; range 0–18); MHC SWB, social well-being subscale (State; range 0–15); SSS-8 = Somatic Symptom Scale-8 (State; range 0–32); VAS, Visual Analogue Scale of well-being (State; range 0–100). Diagonal entries represent generalizability coefficients derived from generalizability theory. Values below the diagonal represent within-person correlations, and values above the diagonal represent between-person correlations. All correlations were statistically significant at *p* < 0.001.

Item-level generalizability coefficients ranged from 0.92 to 0.97, indicating high consistency across persons and measurement occasions (see Table 4).

**Table 4:** Generalizability coefficients, intercorrelations, and descriptive statistics of MHC-state.

Item	*M* (*SD*)	G-coefficient (ICC)	(1)	(2)	(3)	(4)	(5)	(6)	(7)	(8)	(9)	(10)	(11)	(12)	(13)
#1 Happy	2.01 (0.97)	0.93 (0.45)													
#2 Interested in life	1.98 (1.01)	0.95 (0.54)	0.69												
#3 Satisfied with life	1.91 (1.01)	0.95 (0.52)	0.67	0.71											
#4 Social contribution	1.19 (1.08)	0.95 (0.53)	0.42	0.48	0.49										
#5 Social integration	1.62 (1.07)	0.94 (0.50)	0.48	0.50	0.51	0.53									
#6 Social actualization	1.06 (1.02)	0.96 (0.64)	0.38	0.42	0.43	0.50	0.47								
#7 Social acceptance	1.34 (1.07)	0.97 (0.66)	0.40	0.42	0.43	0.43	0.44	0.70							
#8 Social coherence	1.02 (0.99)	0.96 (0.64)	0.35	0.41	0.41	0.47	0.42	0.73	0.63						
#9 Self-acceptance	1.84 (1.03)	0.96 (0.63)	0.46	0.53	0.57	0.45	0.46	0.41	0.42	0.43					
#10 Environmental mastery	1.87 (1.00)	0.92 (0.43)	0.46	0.54	0.53	0.47	0.43	0.34	0.34	0.35	0.53				
#11 Positive relations with others	1.96 (1.00)	0.95 (0.55)	0.49	0.52	0.55	0.44	0.57	0.39	0.41	0.37	0.51	0.49			
#12 Personal growth	1.37 (1.10)	0.96 (0.58)	0.45	0.51	0.46	0.55	0.49	0.50	0.47	0.47	0.50	0.46	0.49		
#13 Autonomy	1.86 (1.04)	0.95 (0.58)	0.44	0.52	0.51	0.46	0.50	0.35	0.35	0.37	0.56	0.49	0.53	0.54	
#14 Purpose in life	1.70 (1.08)	0.97 (0.66)	0.49	0.63	0.66	0.51	0.52	0.50	0.47	0.50	0.62	0.56	0.59	0.59	0.62

MHC-State, mental health continuum-short form (State). G-coefficients were estimated using generalizability theory. ICC values in parentheses. All correlations were statistically significant at *p* < 0.001.

Multilevel reliability analyses further indicated high between-person reliability for the MHC-State scales (*R_kR_* = 0.95–0.97). Within-person reliabilities were lower and varied across dimensions, with the highest estimates for EWB (*R_c_* = 0.70) and MHC-State total score (*R_c_* = 0.68) followed by PWB (*R_c_* = 0.53) and SWB (*R_c_* = 0.41). Floor effects were most pronounced for Items #6 (37.1%; *society is improving*) and #8 (37.6%; *society makes sense*), where the lowest response categories (0 = *never*, 1 = *once*) were frequently selected for these items in daily assessments. Conversely, Item #1 (40.2%; *happy*) and Item #2 (40.9%; *interested in life*) showed ceiling effects, with many responses at the maximum category (3 = *three or more times*).

#### 3.3.2 Multilevel confirmatory factor analysis (MCFA)

The multilevel confirmatory factor analysis indicated acceptable fit at the within-person level (SRMR-W = 0.05). Fit at the between-person level was weaker and slightly exceeded the recommended threshold of 0.09 (SRMR-B = 0.10; Hu and Bentler, 1999). Because SRMR-B can be sensitive to sample characteristics and between-level model complexity, this result should be interpreted with caution (Hsu et al., 2015). The overall CFI was also slightly below the recommended threshold of 0.95 (CFI = 0.93; Hu and Bentler, 1999). As conventional fit indices may be primarily driven by within-level fit, this result should be interpreted together with SRMR-W and SRMR-B.

The additional two-level MCFA based on WLSMV showed a comparable fit pattern, χ^2^(148) = 896.16, *p* < 0.001, RMSEA = 0.04, CFI = 0.93, TLI = 0.92, SRMR-W = 0.04, and SRMR-B = 0.06. Standardized factor loadings (Supplementary Table S4) were moderate to high at both levels, supporting the specified item-factor assignments. At the same time, substantial latent factor correlations, particularly at the between-person level, indicated that the empirical distinction between the subdimensions was limited.

#### 3.3.3 Effects over time

To investigate changes across the daily assessment phase, linear mixed-effects models were computed with day of participation as the Level 1 predictor. Overall well-being (MHC-State) declined slightly but significantly (*b* = −0.14, *SE* = 0.03, *p* < 0.001). An additional multilevel model examined whether retrospective MHC-Trait moderated changes in MHC-State across the daily assessment period. The Day × MHC-Trait interaction was significant (*b* = 0.01, *SE* = 0.002, *p* < 0.001), indicating that participants with higher retrospective person-level well-being showed a less pronounced decline in daily well-being over time. At average levels of MHC-Trait, MHC-State declined slightly over time (*b* = −0.13, *SE* = 0.03, *p* < 0.001). Of the three subscales, emotional well-being showed the strongest decline (*b* = −0.07, *SE* = 0.01, *p* < 0.001), followed by psychological well-being (*b* = −0.04, *SE* = 0.02, *p* = 0.003), whereas social well-being did not show a statistically significant decline (*b* = −0.02, *SE* = 0.01, *p* = 0.053). A null model showed that 73% of the variance in MHC-State was due to differences between individuals (see Supplementary Table S2 for the results of all null models). An item-level analysis of the MHC-State revealed notable variability in stability across the items. ICCs ranged from 0.43 (Item #10: environmental mastery) to 0.66 (Item #7: social acceptance). In an exploratory lagged model, the previous day’s MHC-State score significantly predicted the current MHC-State (*b* = 0.23, *SE* = 0.02, *p* < 0.001), indicating moderate day-to-day stability.

### 3.4 Associations with related retrospective person-level and daily indicators

At the retrospective person-level, retrospective MHC-Trait and its subscales were positively associated with VAS-Trait (*r* = 0.48 to 0.67, all *p* values < 0.001), whereas they were negatively associated with SSS-8-Trait (*r* = −0.27 to −0.42, all *p* values < 0.001). At the daily level, multilevel models showed positive within-person associations between MHC-State and its subscales and VAS-State (*b* = 0.20 to 0.37, all *p* values < 0.001), whereas negative within-person associations emerged between MHC-State and SSS-8-State (*b* = −0.15 to −0.28, all *p* values < 0.001).

## 4 Discussion

The present study investigated whether the Mental Health Continuum-Short Form (MHC-SF; Keyes, 2002, 2005) can be meaningfully adapted to capture both stable and day-to-day aspects of well-being in a German-speaking sample, using a 14-day ESM design. While previous studies have focused primarily on the retrospective person-level applications of the MHC-SF, this study examined its psychometric properties by using a combination of cross-sectional and longitudinal designs. Overall, the findings provide preliminary and qualified support for a daily diary adaptation of the MHC-SF. Evidence was strongest for the total MHC score and emotional well-being, whereas psychological and especially social well-being showed more trait-like properties and weaker evidence for capturing short-term daily fluctuations.

### 4.1 Retrospective person-level well-being

In line with previous findings (e.g., Stieger et al., 2024), the retrospective two-week MHC-SF (MHC-Trait) demonstrated excellent internal consistency, supporting its use as a reliable person-level measure of well-being. The original three-factor model proposed by Keyes (2002, 2005) showed poor fit in CFA in the present sample, which is consistent with previous studies (Jovanović, 2015; Żemojtel-Piotrowska et al., 2018). Accordingly, the CFA results did not provide sufficient support for the original three-factor model when items were restricted to load only on their theoretically assigned factors. In contrast, ESEM provided a substantially better fit than the restrictive CFA models. However, the loading pattern suggested a dominant general well-being factor rather than three distinct person-level dimensions. The additional factors mainly reflected residual structure among selected social well-being items. Thus, although the theoretical distinction between emotional, social, and psychological well-being remains relevant (Rogoza et al., 2018; Żemojtel-Piotrowska et al., 2018), the present findings suggest that the retrospective MHC-Trait structure may be better represented by a flexible multidimensional model with a strong general well-being dimension. An additional second-order CFA showed the same global fit as the correlated three-factor CFA but produced a negative residual variance for psychological well-being. Therefore, the MHC total score was interpreted as an observed composite index of overall positive mental health rather than as evidence for a clearly higher-order latent structure, while subscale-level interpretations, especially for social well-being, should be made cautiously.

At the item level, the most pronounced deviations concerned the social well-being items. In particular, the pattern for items reflecting social integration, social actualization, and social coherence suggested that the SWB dimension may be less clearly differentiated in the present sample. Similar difficulties with the social well-being dimension have been reported in previous validation studies of the MHC-SF (e.g., Santini et al., 2020). Taken together, these findings highlight the need for further refinement of the social well-being dimension and caution against an uncritical adoption of the original factor structure across cultural contexts (Keyes, 2018).

### 4.2 State well-being

The daily adaptation of the MHC-SF (MHC-State) demonstrated acceptable but domain-specific psychometric properties. The strongest evidence emerged for the total MHC score and emotional well-being, whereas psychological and social well-being showed comparatively stronger between-person components and weaker evidence for capturing short-term daily fluctuations. Associations with subjective well-being and somatic symptom burden followed the expected pattern and were broadly consistent with previous ESM research (Stieger et al., 2023b).

The significant Day × retrospective MHC-Trait interaction indicated that individuals with higher person-level well-being showed a less pronounced decline in MHC-State across the daily assessment period. This pattern is consistent with the view that retrospective person-level well-being captures relatively stable between-person differences that are linked to more stable day-to-day well-being patterns and aligns with longitudinal evidence suggesting that a substantial proportion of variance in day-to-day affect reflects stable, trait-like individual differences (Hudson et al., 2017). Multilevel CFA provided partial support for the intended daily measurement structure especially at the within-person level. However, weaker between-person fit, a slightly below-threshold overall CFI, and substantial latent factor correlations in the WLSMV-based model suggest that the empirical distinction between the subdimensions should be interpreted with caution, particularly at the between-person level. Of the three subdomains, emotional well-being (EWB) exhibited the largest proportion of within-person variability (26.4%), underscoring its suitability for daily diary designs.

In contrast, social (SWB) and psychological well-being (PWB) displayed larger between-person variance components, reflecting their more trait-like character (Joshanloo, 2022; van Zyl, 2021). However, it is important to note that ICCs capture only relative proportions of variance and do not represent absolute stability. In the present data, ICC analysis indicated that a substantial proportion of the variance in daily well-being was attributable to differences between individuals, while within-person variability remained meaningful. This pattern suggests that the MHC-State is not equally sensitive to day-to-day fluctuations across all dimensions. Emotional well-being seems to be better suited to daily diary assessments. In contrast, psychological and social well-being appear to be more suitable for capturing person-level differences and longer-term changes than short-term daily fluctuations.

Across the assessment period, emotional and psychological well-being declined slightly, while social well-being did not show a statistically significant decline. This trend may be partly due to methodological influences such as reactivity, habituation, or time-of-day effects (Eisele et al., 2020, 2021). Lagged multilevel analyses indicated moderate autoregressive stability, suggesting that daily well-being was shaped by both situational factors and continuity from one day to the next.

Further exploratory analyses revealed a minor increase in VAS-State over time, whereas MHC-State scores declined slightly. This discrepancy likely reflects differences in the measurement format and construct coverage. Whereas the one-item VAS-State captures a global evaluation of daily well-being on a broad visual analogue scale, the 14-item MHC-State requires broader cognitive reflection across emotional, social and psychological well-being. Accordingly, VAS-State, as a single item, should be regarded as a supplementary indicator rather than a direct replacement for the MHC-State.

### 4.3 Social well-being in daily diary assessment

Although social well-being is theoretically a key component of the MHC-SF, the present results indicated that some of the original social well-being items may be less suitable for daily diary assessment in their current form. Items referring to whether society is improving or whether society makes sense exhibited pronounced floor effects, suggesting that such broad societal evaluations may not be frequently experienced or updated on a day-to-day basis. This does not imply that social well-being is purely trait-like, as longitudinal research indicates that social well-being comprises both stable between-person components and occasion-specific variation (Joshanloo, 2023). However, the present findings suggest that the original MHC-SF social well-being items may be less sensitive to daily fluctuations than emotional well-being items. Moreover, the retrospective person-level ESEM results indicated limited empirical differentiation of the social well-being dimension. Together with the state-level findings, these results suggest that some of the original social well-being items may be too broad and abstract to capture meaningful day-to-day variation in daily diary designs.

### 4.4 Practical implications

This study has several implications for practitioners and researchers interested in measuring well-being in everyday life. First, reducing the number of response categories in the MHC-State proved effective in enabling high compliance during the daily assessment phase, which is in line with previous ESM findings (see Heshmati et al., 2023). Given the deviation from the original scale, future research could examine whether using the full response scale would compromise data quality or participant compliance, and whether this would improve measurement sensitivity (Finstad, 2010).

Second, to ensure the measure’s broader applicability, future studies should investigate how the items perform in different populations and longitudinal studies. This is particularly important for social well-being items, as their functioning may vary with cultural or contextual factors (Santini et al., 2020; Żemojtel-Piotrowska et al., 2018). In this study, some MHC-State items from the psychological and social well-being domains showed floor effects, suggesting that these aspects were reported infrequently in daily life. For SWB, this may indicate that the items did not fully cover the construct, or that this dimension was less present in participants’ everyday lives. Subsequent studies should therefore examine whether the reduced usage of these items is due to factors associated with their content or context.

Finally, of the three MHC-SF well-being dimensions, emotional well-being was found to be the most dynamic, indicating its high sensitivity to situational influences, making it well-suited for ESM applications. This highlights its potential for use in personalised, time-sensitive interventions to enhance, or monitor individuals’ well-being in applied settings.

### 4.5 Limitations

Although the study aimed to include a diverse community sample, the results cannot readily be generalized to the broader population. Furthermore, both self-selection and the requirement to use a smartphone device may have resulted in a sampling bias. Nevertheless, the achieved sample size and intensive longitudinal design provided sufficient variability for the present analyses.

A further limitation concerns the use of external indicators. Subjective well-being was assessed using a single-item visual analogue scale (VAS) and associations with somatic symptom burden represented only one external criterion. These associations should be interpreted with caution, given that the generalisability of the SSS-8-State was low in the present daily assessment context. Future studies should include a broader range of indicators, such as positive and negative affect, life satisfaction, stress and other mental health outcomes, as well as established daily well-being measures (e.g., mPERMA; Heshmati et al., 2023).

Another limitation refers to the measurement design. The study employed an interval-contingent daily diary design with one end-of-day assessment per day, capturing retrospective summaries of daily experiences rather than momentary states. Although end-of-day reports have been shown to approximate aggregated momentary assessments for some constructs (Broderick et al., 2009), they may still be influenced by recall processes such as recency or peak-end effects. Accordingly, the present findings should be interpreted at the level of day-to-day well-being rather than momentary experience. Future studies using event-contingent designs are needed to examine whether the observed psychometric properties generalize to real-time assessments.

Finally, given the availability of alternative questionnaires (see VanderWeele et al., 2020 for a review), it remains unclear whether the MHC-SF is the most suitable instrument for daily assessments. However, its strong theoretical foundation, extensive validation, multidimensional structure and cross-cultural applicability support its suitability for capturing both hedonic and eudaimonic aspects of well-being (Hone et al., 2014; Joshanloo, 2022; Lamers et al., 2011). This is also consistent with recent evidence from item pool visualization analyses (IPV, Dantlgraber et al., 2019), which showed that well-being questionnaires differ substantially in their construct coverage and the extent to which they capture distinct facets of well-being (Ploke et al., 2024). Notably, the MHC-SF assesses emotional, psychological, and social well-being with a single concise measure, whereas other well-being measures vary considerably in the dimensions they cover, and do not always include social well-being (Linton et al., 2016). Including social well-being as a core component is important because it captures how individuals perceive their functioning in relation to society and community (Keyes, 1998). Consequently, the MHC-SF is particularly relevant for ESM designs that aim to capture multiple facets of well-being in everyday life.

### 4.6 Conclusion

The present study provides preliminary and qualified evidence that the MHC-SF can be adapted for use in daily diary research. However, this support was not consistent across all dimensions. The daily adaptation appeared to be most suitable for the total score and emotional well-being. In contrast, psychological and social well-being showed stronger between-person components and weaker evidence for capturing day-to-day fluctuations. Of the three dimensions, emotional well-being (EWB) emerged as the most dynamic, making it particularly well-suited to ESM approaches and personalised, time-sensitive interventions, such as mobile applications for tracking well-being (Heshmati et al., 2023).

In summary, the MHC-State may help bridge the gap between traditional self-report measures and modern, experience-based approaches. Its brevity (14 items) and multidimensional coverage of emotional, psychological, and social well-being make it a promising instrument for assessing overall and emotional aspects of daily well-being. The findings also point to important opportunities for further refining the psychological and social well-being dimensions to better capture short-term fluctuations in intensive longitudinal designs.

## Data Availability

The datasets presented in this study can be found in online repositories. The names of the repository/repositories and accession number(s) can be found at: https://osf.io/y3f6g.
