# Supplementary material for: Capturing daily well-being: psychometric evaluation of the MHC-SF in a daily diary design
Source: Front Psychol. 2026 Jul 28;17:1887563. doi: 10.3389/fpsyg.2026.1887563 (PMC13457135; doi:10.3389/fpsyg.2026.1887563)
Supplement: Supplementary file 1 [file Table_1.DOCX]

Supplementary Material

Capturing daily well-being: Psychometric evaluation of a daily diary adaptation of the MHC-SF

# Supplementary Figures and Tables

**Figure S1**

*Screenshots of personalized and general graphical feedback in ESMira*

General feedback across all participants

Individual feedback for the respective participant

**Table S1**

*Descriptive Statistics and Intercorrelations of MHC-Trait Scores*

| Item # | *M* (*SD*) | 01 | 02 | 03 | 04 | 05 | 06 | 07 | 08 | 09 | 10 | 11 | 12 | 13 |
| --- | --- | --- | --- | --- | --- | --- | --- | --- | --- | --- | --- | --- | --- | --- |
| MHC 01 | 3.74 (0.99) |  |  |  |  |  |  |  |  |  |  |  |  |  |
| MHC 02 | 3.64 (1.23) | .69 |  |  |  |  |  |  |  |  |  |  |  |  |
| MHC 03 | 3.53 (1.27) | .72 | .81 |  |  |  |  |  |  |  |  |  |  |  |
| MHC 04 | 2.86 (1.37) | .44 | .54 | .52 |  |  |  |  |  |  |  |  |  |  |
| MHC 05 | 3.42 (1.33) | .60 | .55 | .53 | .59 |  |  |  |  |  |  |  |  |  |
| MHC 06 | 2.26 (1.56) | .41 | .44 | .47 | .50 | .43 |  |  |  |  |  |  |  |  |
| MHC 07 | 2.53 (1.64) | .39 | .43 | .45 | .52 | .41 | .80 |  |  |  |  |  |  |  |
| MHC 08 | 2.10 (1.60) | .40 | .44 | .45 | .48 | .38 | .89 | .75 |  |  |  |  |  |  |
| MHC 09 | 3.57 (1.40) | .60 | .64 | .63 | .43 | .45 | .47 | .43 | .48 |  |  |  |  |  |
| MHC 10 | 3.54 (1.20) | .58 | .65 | .66 | .50 | .51 | .41 | .36 | .41 | .61 |  |  |  |  |
| MHC 11 | 3.93 (1.18) | .53 | .59 | .58 | .48 | .67 | .39 | .38 | .37 | .47 | .63 |  |  |  |
| MHC 12 | 3.08 (1.46) | .49 | .57 | .51 | .55 | .48 | .53 | .53 | .51 | .61 | .51 | .48 |  |  |
| MHC 13 | 3.60 (1.32) | .49 | .56 | .54 | .48 | .45 | .30 | .28 | .32 | .54 | .47 | .49 | .56 |  |
| MHC 14 | 3.33 (1.53) | .61 | .75 | .75 | .54 | .52 | .49 | .47 | .47 | .68 | .68 | .58 | .58 | .64 |

*Note.* MHC = Mental Health Continuum SF Trait; α = .94.

**Table S2**

*Results of the Null Models for MHC-State and its Subscales*

| Predictor | *B* | *SE* | *t*-value | *ICC* |
| --- | --- | --- | --- | --- |
| MHC – overall score |  |  |  |  |
| Intercept | 22.84 | 0.56 | 40.61*** | .73 |
| Subscale PWB |  |  |  |  |
| Intercept | 10.63 | 0.26 | 41.10*** | .70 |
| Subscale SWB |  |  |  |  |
| Intercept | 6.26 | 0.22 | 28.46*** | .71 |
| Subscale EWB |  |  |  |  |
| Intercept | 5.93 | 0.13 | 46.20*** | .57 |

*Note.* ICC = Intraclass correlation coefficient, MHC State = Mental Health Continuum-SF State, EWB = Mental Health Continuum Emotional Well-being State, PWB = Mental Health Continuum Psychological Well-being State; SWB = Mental Health Continuum Social Well-being State. *** *p* < .001

**Table S3**

*Standardized ESEM factor loadings for the retrospective two-week MHC-SF*

| Dimension | Item # | F1 | F2 | F3 |
| --- | --- | --- | --- | --- |
| EWB | #1 Happy | **.83** | -.11 | .05 |
| EWB | #2 Interested in life | **.88** | -.05 | -.06 |
| EWB | #3 Satisfied | **.92** | -.09 | -.10 |
| SWB | #4 Contribution to society | **.68** | .19 | .14 |
| SWB | #5 Belonging to a community | .68 | .03 | .**73** |
| SWB | #6 Society improving | .58 | .**79** | .02 |
| SWB | #7 People are good | **.59** | **.59** | .02 |
| SWB | #8 Society makes sense | .60 | **.69** | -.04 |
| PWB | #9 Liked most parts of personality | **.82** | .03 | -.11 |
| PWB | #10 Managing daily responsibilities | **.81** | -.08 | -.02 |
| PWB | #11 Warm and trusting relationships | **.76** | -.03 | .24 |
| PWB | #12 Experiences for growth | **.75** | .14 | -.01 |
| PWB | #13 Confidence in own ideas | **.72** | -.05 | .00 |
| PWB | #14 Life has meaning | **.90** | .02 | -.12 |

*Note.* EWB = emotional well-being; SWB = social well-being; PWB = psychological well-being. Values are standardized factor loadings from the three-factor ESEM model with geomin rotation. The highest loading per item is shown in bold. Factor correlations were small: F1–F2 = .05, F1–F3 = .07, and F2–F3 = -.03.

**Table S4**

*Standardized factor loadings from the WLSMV-based two-level MCFA of the MHC-State*

| Dimension | Item # | Within-level | Between-level |
| --- | --- | --- | --- |
| EWB | #1 Happy | .82 | .86 |
| EWB | #2 Interested in life | .83 | .95 |
| EWB | #3 Satisfied with life | .83 | .95 |
| SWB | #4 Social contribution | .61 | .89 |
| SWB | #5 Social integration | .66 | .87 |
| SWB | #6 Social actualization | .69 | .76 |
| SWB | #7 Social acceptance | .64 | .74 |
| SWB | #8 Social coherence | .63 | .75 |
| PWB | #9 Self-acceptance | .67 | .82 |
| PWB | #10 Environmental mastery | .59 | .85 |
| PWB | #11 Positive relations with others | .66 | .84 |
| PWB | #12 Personal growth | .65 | .83 |
| PWB | #13 Autonomy | .64 | .79 |
| PWB | #14 Purpose in life | .68 | .95 |

*Note*. Standardized factor loadings are STDYX estimates from the WLSMV-based two-level multilevel confirmatory factor analysis. EWB = emotional well-being; SWB = social well-being; PWB = psychological well-being. All loadings were statistically significant at *p* < .001.
